# A Multistage Hemiplegic Lower-Limb Rehabilitation Robot: Design and Gait Trajectory Planning

**DOI:** 10.3390/s24072310

**Published:** 2024-04-05

**Authors:** Xincheng Wang, Hongbo Wang, Bo Zhang, Desheng Zheng, Hongfei Yu, Bo Cheng, Jianye Niu

**Affiliations:** 1 Hebei Provincial Key Laboratory of Parallel Robot and Mechatronic System, Yanshan University, Qinhuangdao 066004, China; wangxincheng@stumail.ysu.edu.cn (X.W.); hongbo_w@ysu.edu.cn (H.W.); 15505026028@163.com (B.Z.); chaoxingleishen@126.com (D.Z.); 2 School of Mechanical Engineering, Yanshan University, Qinhuangdao 066004, China; 3 Academy for Engineering & Technology, Fudan University, Shanghai 200433, China; 4 School of Arts and Design, Yanshan University, Qinhuangdao 066004, China; 5 Hebei Technology Innovation Center for Intelligent Industrial Design, Qinhuangdao 066004, China; 6 Qinhuangdao Hospital of Traditional Chinese Medicine, Qinhuangdao 066099, China; barrycb@163.com

**Keywords:** exoskeletons, lower limb rehabilitation robot, multiple recovery stages, hemiplegia, gait trajectory planning

## Abstract

Most lower limb rehabilitation robots are limited to specific training postures to adapt to stroke patients in multiple stages of recovery. In addition, there is a lack of attention to the switching functions of the training side, including left, right, and bilateral, which enables patients with hemiplegia to rehabilitate with a single device. This article presents an exoskeleton robot named the multistage hemiplegic lower-limb rehabilitation robot, which has been designed to do rehabilitation in multiple training postures and training sides. The mechanism consisting of the thigh, calf, and foot is introduced. Additionally, the design of the multi-mode limit of the hip, knee, and ankle joints supports delivering therapy in any posture and training sides to aid patients with hemiplegia in all stages of recovery. The gait trajectory is planned by extracting the gait motion trajectory model collected by the motion capture device. In addition, a control system for the training module based on adaptive iterative learning has been simulated, and its high-precision tracking performance has been verified. The gait trajectory experiment is carried out, and the results verify that the trajectory tracking performance of the robot has good performance.

## 1. Introduction

Lower limb rehabilitation training after stroke is a key means to restore the walking ability and daily self-care abilities of hemiplegic patients [1,2]. The Brunnstrom theory divides the rehabilitation of stroke patients into six recovery stages [3]. The rehabilitation of patients with hemiplegia is diverse, including multiple training postures and uncertain affected sides. Traditional physical therapy, which mainly relies on manual intervention, can effectively address various rehabilitation situations for patients with hemiplegia [4]. However, due to the high labor intensity and the high demand for rehabilitation therapists, the research and application of lower limb rehabilitation robots are receiving increasing attention [5].

The lower limb rehabilitation robot, which can only perform lying posture training, has a greater limitation and is more suitable for stroke patients in the early recovery stage. Supine [6] is a gait training device installed on the bed, composed of metal links of the thigh, calf and ankle orthosis. The flexion and extension motions of each joint are driven by a linear actuator. It can only perform dual-leg training with a specially designed bed. It was designed for rehabilitation with either the left or right training side in both the sitting and lying positions. HipBot [7], developed by Zacatecas Political University in Mexico, is an end-traction hip-joint lower limb rehabilitation robot. It has five degrees of freedom and can perform rehabilitative training, including abduction/adduction and flexion/extension. Additionally, HipBot offers rehabilitation on both right and left legs (individually) in the lying position. The Biofeedback Therapeutic-Exercise-Supporting Manipulator [8], designed by Japan Mie University, provides isokinetic motion of the knee and hip joints in the sagittal plane. It is an end-traction lower limb rehabilitation robot, installed in the middle of a specially designed bed, so it can achieve single-leg rehabilitation training for the left or right leg.

Sitting and lying lower limb rehabilitation robots are suitable for stroke patients in both the early and middle stages of recovery, which can be divided into exoskeleton types and end-traction types. The exoskeleton style can better correspond to human joints. Motion Maker [9,10], developed by the Swortec company, is an exoskeleton-type multi-position lower limb rehabilitation robot. Both the left and right mechanical legs have three degrees of freedom in flexion, extension, and rotation of the hip, knee, and ankle. Similarly, LLR-Ro [11] and iLEG [12] are also exoskeleton lower limb rehabilitation robots that adopt similar structures. They include both lying and sitting rehabilitative postures with only dual-leg training. Physiotherabot [13] and ViGRR [14] include not only sagittal movements but also hip joint adduction and abduction movements. In addition, they can achieve rehabilitation for one or both legs. However, during single-leg training, a single-leg mechanism does not have the ability to swap to the other side. The end-traction type is more in line with the physician’s dragging technique. TEM LX2 typeD [15] is a multi-posture end-traction lower limb rehabilitation robot developed by Japan Yaskawa.

The tilting bed lower limb rehabilitation robot combines the tilting bed and the lower limb rehabilitation device to achieve lower limb rehabilitation training in both lying and standing positions. Except for training in the sitting position, it almost covers lower limb training for stroke patients in all stages of recovery. Erigo [16] is used for progressive verticalization and cyclic leg movements, combined with synchronized leg muscle FES and weight load to ensure safe and stable upright positions. Its training involves gradually verticalizing and applying continuous step-like movements to subject the patient’s lower limbs to various movement patterns and loads. Nukawa [17,18] consists of two legs, each consisting of a three-link mechanism and an electronic position and force control, with each leg having three degrees of freedom (3DOF). This design also features a brushless DC motor, a power driver, and a position sensor to execute control strategies that can generate multiple rehabilitation modes. They can both achieve lower limb rehabilitation training in a lying or standing position. However, they can only conduct bilateral leg training instead of unilateral leg training, and patients need to move from the bed to the tilting bed, which can be time-consuming and effortful.

Gait rehabilitation robots mainly achieve lower limb rehabilitation training in standing posture, making them suitable for stroke patients in the later stages of recovery. It includes treadmill-gait rehabilitation robots and overground-gait rehabilitation robots. Lokomat [19] consists of a treadmill, a dynamic unloading system, and two lightweight robot actuators connected to the legs of the subjects. The hip and knee joints are driven by small DC motors and linear ball screw components. The motion trajectory of Lokomat is completely programmable and can be adjusted according to each person’s body shape and step size preferences. LOPES [20,21] consists of a bilateral exoskeleton rehabilitation robot located above an instrumented treadmill. It is lightweight, and its impedance is controlled by a Bowden cable-driven series elastic actuator. The exoskeleton provides a freely translatable pelvis that drives lateral and forward/backward movement. In addition, it includes two driving rotation axes on the hip and knee (abduction/adduction of the hip and flexion/extension of the hip and knee). ALEX [22,23] and LokoHelp [24,25] are also treadmill gait rehabilitation robots. The WalkTrainer [26] is a verticalized robot for over-ground walking recovery. It is equipped with leg and pelvic orthotics, active weight support, and a mobility frame, allowing users to train in large corridors and other areas. NaTUre-gaits [27] has introduced three robot modules, enabling the robot gait rehabilitation system to provide weight support for gait rehabilitation in the background of ground walking and to assist in hip and lower limb movements. The unique feature of NaTure gait is that it provides pelvic motion assistance and weight support through pelvic assistive mechanisms. Other overground gait rehabilitation robots include eLEGS [28], HAL [29] and ACPGs [30].

These lower-limb rehabilitation robots have been developed to provide single-legged or dual-legged rehabilitation therapy in either the early or late stages of recovery. In addition, trajectory tracking methods [31,32,33] are also widely used in these rehabilitation robots. However, there is currently no lower limb rehabilitative device that can provide rehabilitation to patients with hemiplegia in all stages of recovery. A lower limb rehabilitation robot that can provide therapy in all postures, including lying, sitting, and standing, would be particularly useful in cases of hemiplegia, as rehabilitation is required for all stages of recovery and could be implemented with a single device. Therefore, this research has the following highlights: (1) A multistage hemiplegic lower limb rehabilitation robot with a compact structure, convenient movement, low manufacturing cost, and adjustable mechanical leg has been designed, which can be combined with the bed, seat, and suspension mechanism to carry out rehabilitation training in lying, sitting, and standing positions. (2) A gait trajectory planning strategy based on the motion capture device (Xsens MVN Link) is designed to reconstruct the patients’ walking ability. And an adaptive trajectory tracking controller is used to provide more effective rehabilitation training for patients.

This paper describes the design, the gait trajectory planning, the adaptive trajectory tracking controller, and the experiment of a novel multistage hemiplegic lower-limb rehabilitation robot (MHLRR) for patients with hemiplegia in all stages of recovery. The MHLRR has designed multiple limit modes for joints to suit different modes of therapy. The gait trajectory planning is completed based on the motion capture device called the Xsens MVN Link. In addition, an adaptive trajectory tracking controller is applied to track the gait trajectory. Finally, the experiment has been completed and proved that the MHLRR is able to implement the planned gait trajectory.

## 2. Mechanical Design

The multistage hemiplegic lower-limb rehabilitation robot (MHLRR) is an exoskeleton type that can be used to conduct motor rehabilitation of the lower limb for patients with hemiplegia in multiple recovery stages. It has several features, including multistage usability, bi-side usability (either left or right training side), compact structure design, mechanically adjustable limits and easy mobility.

### 2.1. Overall Structure

**Figure 1:**
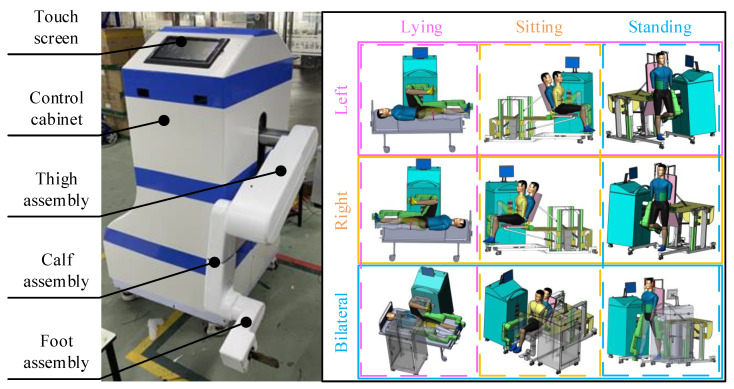
Prototype of the MHLRR. MHLRR: Multistage Hemiplegic Lower-Limb Rehabilitation Robot. Multiple training postures and training sides for patients with hemiplegia in all stages of recovery.

The overall structure mainly consists of the touch screen, the control cabinet, the thigh assembly section, the calf assembly section and the foot assembly section, as shown in Figure 1. The leg orthosis can complete the rehabilitation of three joints, including the hip, knee, and ankle, in the sagittal plane. It has a thigh length adjustment range of 400 mm to 500 mm and a calf length adjustment range of 320 mm to 420 mm, which can adapt to most patients with hemiplegia. The height of the leg orthosis adjustment range of 540~1040 mm allows for training in a lying position with a common bed, training in a sitting position with a seat, and training in a standing position with a suspension mechanism. The robot is fitted with four universal wheels on its underside that can be easily moved around to facilitate coordination with assistive devices to meet the needs of patients in multiple stages of recovery. The structure of the MHLRR can meet the rehabilitative needs of patients with hemiplegia, while it can switch to either the left or right training side.

### 2.2. Thigh Assembly

**Figure 2:**
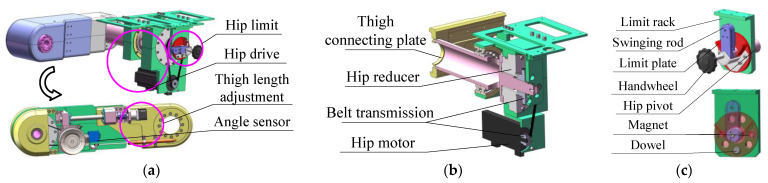
The thigh assembly of the MHLRR: (**a**) The overall structure of the thigh; (**b**) The transmission structure of the hip joint; (**c**) The limiting structure of the hip joint, which includes five limiting modes for three training postures and two training sides.

As shown in Figure 2a, the thigh structure mainly includes the hip joint drive mechanism, the hip joint limit mechanism, the angle sensor, and the thigh length adjusting mechanism. Among them, the thigh length adjusting mechanism mainly consists of the leg length adjusting motor and the linear position sensor (KTC2-100 mm, by Miran Technology Co., Ltd., Shenzhen, China). As shown in Figure 2b, the hip motor module (SMP8024B, by Shanghai Mindong Mechanism Electron Co., Ltd., Shanghai, China) is arranged on the frame below the hip rotation axis, driving the thigh connecting plate to rotate through the T-shaped timing belt and the harmonic reducer (LHSG-40-100-C-III, by Suzhou Shiyue Transmission Technology Co., Ltd., Suzhou, China) to achieve hip movement. Before training, the angle sensor (LVT416T, 0~±180°, by Msensor Technology Co., Ltd., Wuxi, China) provides feedback on the initial angle of the thigh when it is reset. During training, the motor encoder provides real-time feedback on the angle and angular velocity of the hip joint.

As shown in Figure 2c, based on the human lower limb hip mobility in a variety of situations, the limit rack is designed with 5 slots corresponding to the 5 mechanical limiting modes of the hip joint. The V-shaped baffle on the limit plate is designed to be 80°. The swinging rod is fixed to the hip joint pivot, and the limit plate is fixed to the limit handwheel. The overload protection of the joint motor is triggered by the contact between the limit plate and the swinging rod, which provides safety protection when the hip joint moves to its limit position. In this case, the dowel limits the relative rotation of the limit plate and the limit rack, and a pair of magnets are used to attract the limit rack and the limit plate. The five mechanical limits of the hip joint can be switched by pulling out the handwheel to separate the limit plate from the limit rack, then rotating the handwheel to align the dowel with the slots in the limit rack, and finally pushing in the handwheel to make the limit plate adsorbed to the limit rack by magnetic suction.

### 2.3. Calf Assembly

**Figure 3:**
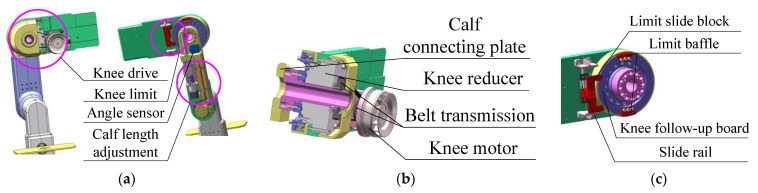
The calf assembly of the MHLRR: (**a**) The overall structure of the calf; (**b**) The transmission structure of the knee joint; (**c**) The limiting structure of the knee joint, which includes two limiting modes for two training sides.

As shown in Figure 3a, the calf structure mainly contains the knee joint drive mechanism, the knee joint limiting mechanism, the angle sensor and the calf length adjusting mechanism. Among them, the calf length adjusting mechanism is driven by the leg length adjusting motor, which realizes the adjustment of the calf length by making the upper calf connecting plate and the lower calf connecting plate. They are fixed on the slide slider, respectively, and make a relative movement along the calf direction. The calf length of the patient is analyzed and recorded with the aid of a voltage signal from a linear position sensor (KTC2-100 mm, by Miran Technology Co., Ltd., Shenzhen, China).

As shown in Figure 3b, the knee motor module (NBL9040, by Shanghai Mindong Mechanism Electron Co., Ltd., Shanghai, China) drives the upper calf attachment plate to rotate for knee motion via a T-shaped timing belt and a harmonic reducer (LHSG-32-100-C-III, by Suzhou Shiyue Transmission Technology Co., Ltd., Suzhou, China). Before training, the angle sensor (LVT416T, 0~±180°, by Msensor Technology Co., Ltd., Wuxi, China) provides feedback on the initial angle of the calf when it is reset.

As shown in Figure 3c, the overload protection of the knee motor is triggered by the contact between the limit slide block and the limit baffle to achieve safety protection when the knee joint moves to the limiting position. Considering the two training sides of the hemiplegic patients, the knee-limiting mechanism is designed to be switchable and contains two limiting modes. When the limit slide block is slid to the top of the slide rail, the right knee training mode is selected, with a mechanical limiting range of (−140° to 0°). When the slide limit block is slid to the bottom of the slide rail, the left knee training mode is selected, with a mechanical limit range of 0° to +140°.

### 2.4. Foot Assembly

**Figure 4:**
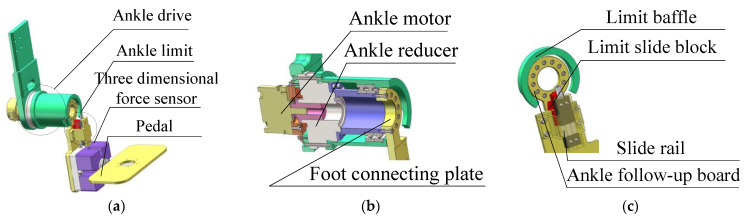
The foot assembly of the MHLRR: (**a**) The overall structure of the foot; (**b**) The transmission structure of the ankle joint; (**c**) The limiting structure of the ankle joint, which includes two limiting modes for two training sides.

As shown in Figure 4a, the foot structure mainly comprises the ankle driving mechanism, the ankle limiting mechanism, the three-dimensional force sensor (R121C, by Changzhou Right Measurement and Control System Co., Ltd., Changzhou, China), and the foot pedal. As shown in Figure 4b, considering the lightweight and compactness of the foot structure, the ankle motor module (EC60flat, by Maxon motor ag, Sachseln, Switzerland) is mounted on the calf plate, co-axial with the ankle joint, and transmits motion to the pedal joint via a harmonic reduction gear (LHSG-20-100-C-III, by Suzhou Shiyue Transmission Technology Co., Ltd., Suzhou, China). Motor encoders are used to give feedback on ankle joint angle and angular velocity. Additionally, the three-dimensional force sensors are mounted on the side of the pedal to measure the human–machine interaction forces at the end of the lower limb. The torque of the hip, knee and ankle joints is obtained by collecting voltage signals in the three dimensions in the sagittal plane and converting them into force signals.

As shown in Figure 4c, the overload protection of the ankle motor is triggered by the contact between the limit slide block and the limit baffle, enabling safety protection when the ankle joint moves to the limit position. The limiting mechanism is designed to be switchable depending on the training side of the patients with hemiplegia. The ankle joint limit in the right leg training mode is selected when the limit slide block is slid to the top, with a mechanical limit range of −30° to +45°. Moreover, the ankle joint limit in the left leg training mode is selected when the limit block is slid to the bottom of the slide, with a mechanical limit range of −45° to +30°.

## 3. Gait Trajectory Planning Based on the Gait Models

### 3.1. Gait Trajectory Collection

**Figure 5:**
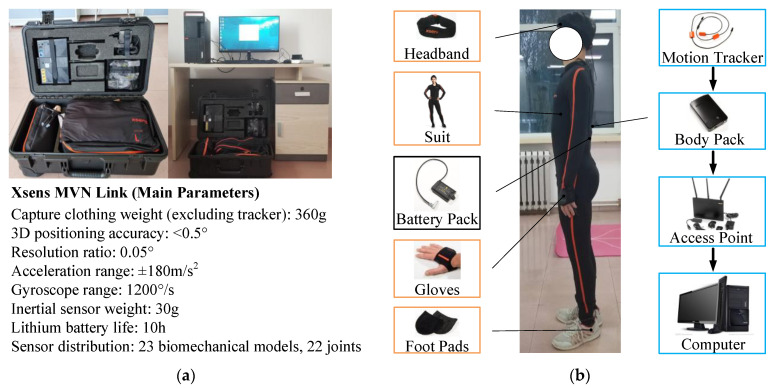
The motion capture acquisition device: Xsens MVN Link. (**a**) The profile and detailed parameters; (**b**) The participant wearing the device and the transmission process of the signal.

Within the context of the MHLRR’s functionality and design, the Xsens MVN Link (by Xsens Technologies B.V., Enschede, The Netherlands) can be used to track and analyze user movements, aiding in the creation of gait trajectory plans that are more biomechanically congruent with human kinematics. Xsens MVN Link is an advanced motion capture (MoCap) hardware system that is designed to accurately capture human body movement data. This system typically includes a series of Inertial Measurement Units (IMUs), which record movements through sensors attached at key positions on the user’s body. Figure 5a shows its main parameters. It is particularly important during the testing and optimization process for the drive component parameters of lower limb joints, including the hip, knee, and ankle. Data provided by Xsens MVN Link can be used to simulate and evaluate movement performance, ensuring that joint actuation aligns with the natural characteristics of human gait. Motion trackers placed at 13 key body parts capture the gait trajectory signals and then transmit through the body pack and the access point, finally reaching the user’s endpoint device, as shown in Figure 5b.

**Figure 6:**
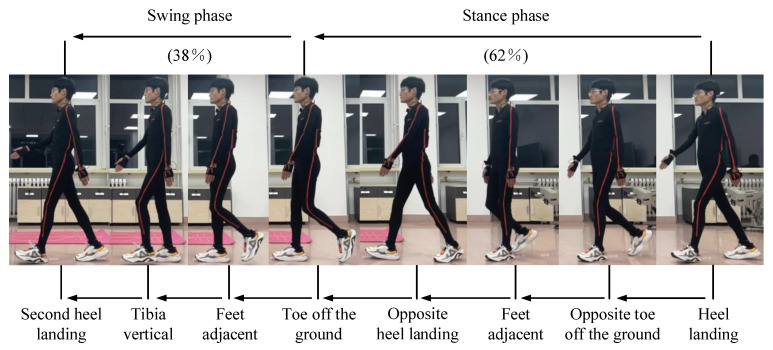
A complete gait cycle of the participant.

The Xsens MVN Link is used to collect kinematic data of the human lower limb joints to obtain the original gait characteristic trajectories of the walking process. The length parameters of various body parts of the gait data collector are shown in Table 1. Figure 6 shows the complete gait cycle of the participant, with the left foot chosen as the subject of study. The stance phase, where the left foot contacts the ground and bears the weight, can be further subdivided into five sub-phases, including heel landing, opposite toe off the ground, feet adjacent, opposite heel landing, and toe off the ground. Moreover, the swing phase, where the left foot leaves the ground and swings until it contacts the ground again, can be subdivided into four sub-phases, including the toe off the ground, feet adjacent, tibia vertical, and second heel landing. Additionally, the stance phase and swing phase, respectively, account for 62% and 38% of the gait cycle phase.

**Figure 7:**
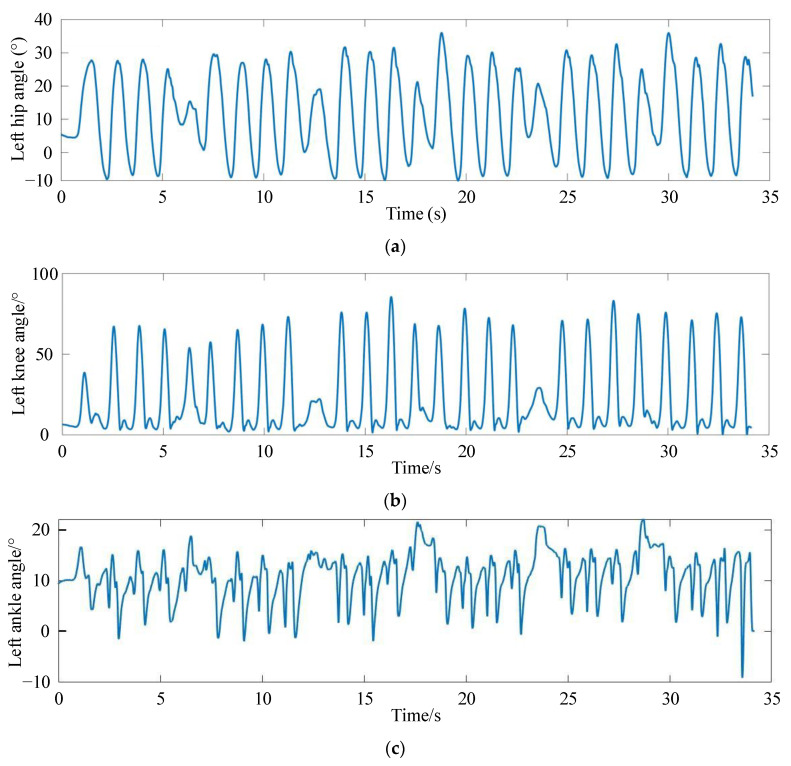
The gait motion trajectories collected from the participant. (**a**) The gait motion trajectories of the left hip joint; (**b**) The gait motion trajectories of the left knee joint; (**c**) The gait motion trajectories of the left ankle joint.

The kinematic data of the lower limb joints during normal walking of the subject were collected by the Xsens MVN Link with a frame rate of 240 fps, which effectively characterizes the motion patterns of walking. Figure 7a–c show the gait trajectories of the hip, knee, and ankle joints on the left side, respectively. We can find that the angular displacement of the hip joint, knee joint, and ankle joint changes periodically, but there is a certain deviation between each period. At the same time, the motion range of the left hip joint is from −10° to 35°, the knee joint from 0° to 80°, and the ankle joint from 0° to 20°. It verifies that the collected gait data exhibit characteristics such as stability, periodicity, and coordination. On this basis, a foundation is established for deducing and studying the gait trajectory within a single period.

### 3.2. Periodic Information Extraction

**Figure 8:**
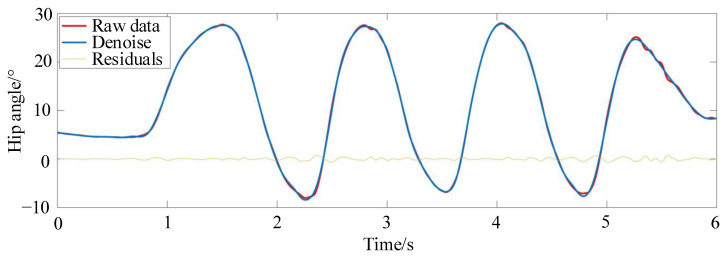
The preprocessed data of the hip angle.

The gait trajectory data collected cannot be used directly for gait trajectory planning because it is discrete and unordered. Therefore, data preprocessing is required for the discrete data points. Through multi-layer iterative filter decomposition and reconstruction, the preprocessing method of Symlet wavelets can realize signal decomposition and reconstruction on different frequency band scales, and it is suitable for signal compensation and filtering processing. Therefore, Symlet wavelets are used to preprocess the collected joint motion information to reduce noise errors in the acquisition channels. The signal-to-noise ratio after noise reduction is 36.3996, and the root mean square error value is 0.25. Symlet wavelets are quite sensitive to very large and very small errors, reflecting a good noise reduction effect. It effectively reduces phase distortion when analyzing and reconstructing the signal, and the preprocessed data on hip angle are shown in Figure 8.

**Figure 9:**
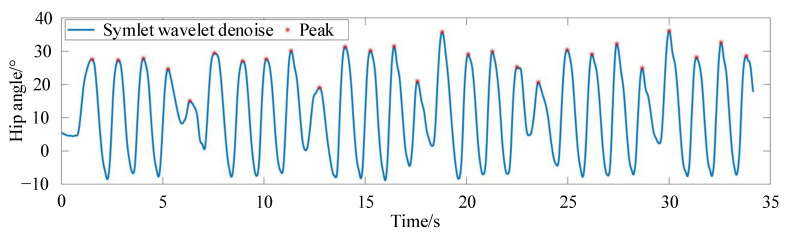
The peak-finding positions of the hip joint after wavelet denoising.

The key periodic information in gait joint motion data lies in the peaks and troughs. Due to the complex structure of the collected data, we use the first derivative to locate the high-positioned peaks from the collected data and then a cubic function derivative to determine if there are any missed peaks. At the same time, we select the appropriate sensitivity to perform a secondary area-check for the known peaks to distinguish closely situated multiple peaks and spurious peaks. Figure 9 shows the peak-finding positions of the gait trajectory of the hip joint after wavelet denoising.

**Figure 10:**
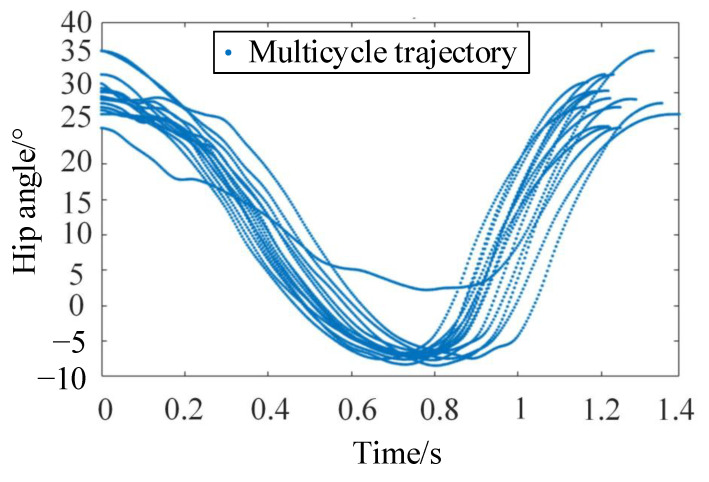
The extractions of hip joint angular displacement for 18 sets of individual cycles.

The acquisition of the standard gait trajectory cyclic function is a prerequisite for precise gait trajectory planning. Using the extracted data peak points, extractions of joint angular displacement for 18 sets of individual cycles are performed, and the hip angle data is shown in Figure 10. Due to factors related to the subject as well as the external disturbances, for the same gait trajectory’s multiple cyclic function models, phenomena such as slight swaying left and right, unequal cycle times, and uneven stride lengths occur, inevitably leading to a large random angular displacement error.

### 3.3. BP Neural Network Calibration

**Figure 11:**
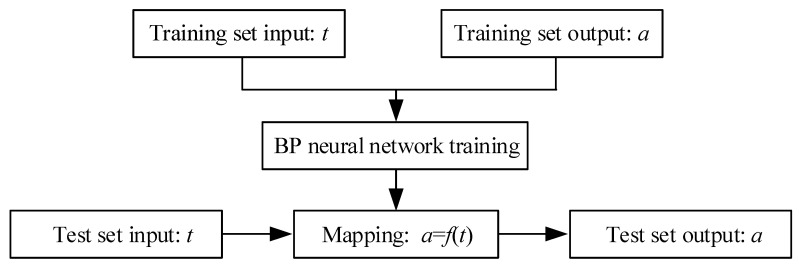
BP neural network model.

To address the mentioned issues, a calibration fitting function based on the BP neural network is used. By analyzing the regression relationship model between the multiple sample cycle times *t* and the joint angular displacement, we have established a mapping relationship. The joint angular displacement information is calibrated online using the time parameter of *t*, as shown in Figure 11.

For the acquired gait trajectory, multiple periodic functions of the same trajectory are divided into test and training sets. The cycle with sequence number 1 is designated as the test set, while the cycles with sequence numbers 2 to 18 are allocated to the training set, as shown in Table 2.

**Figure 12:**
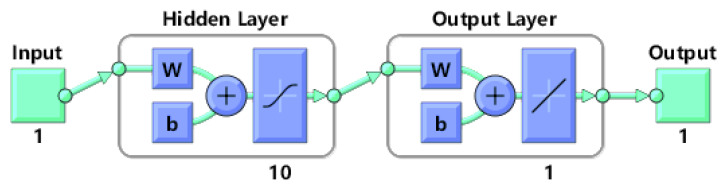
Three-layer BP neural network.

The three-layer BP neural network consists of an input layer, an output layer, and a hidden layer. Multi-cycle gait trajectories are fed into the input layer, then mapped one by one through the hidden layer before being transmitted to the output layer. After error analysis, the errors are backpropagated to modify weights and thresholds, as shown in Figure 12. Based on the structure of the cyclic gait data, the number of nodes in both the input and output layers is 1. The number of neurons in the hidden layer is determined to be 10 by trial and error. Error description parameters such as MAE, MSE, and RMSE are used to evaluate the test, and the results are shown in Table 3.

**Figure 13:**
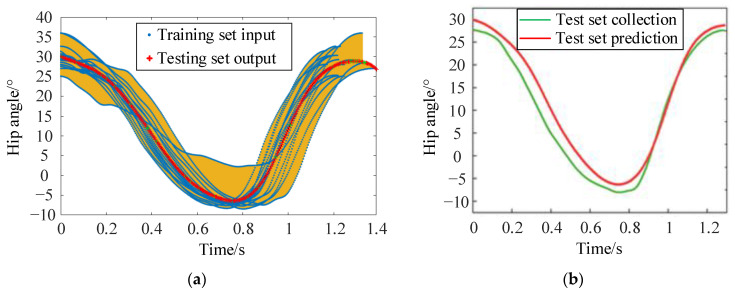
(**a**) Training set fitting simulation; (**b**) Test set fitting simulation.

Figure 13a shows the fitting simulation of the training set and the testing set of the hip joint. The yellow background represents the range of all training set inputs. The testing set has evaluated the model performance of the joint angular displacement and the cyclic time *t*. Figure 13b shows the testing set fitting simulation, which indicates that the selected bp neural network model fits well and has high accuracy.

### 3.4. Fourier Function Curve Fitting

Based on the above, the motion trajectory information of each joint can be approximately regarded as a periodic function. The Fourier function available in MATLAB R2022a’s Curve Fitting Toolbox was used for function fitting, and a mathematical model of gait time trajectory was established. According to the fitting effect characterization coefficients in Table 4, the R-square values of the decision coefficients for the fitted correction of each joint angle displacement are all close to 1, satisfying the fitting accuracy requirements. Thus, it can provide accurate position information for real-time control of joint angle displacement, including hip, knee, and ankle.

**Figure 14:**
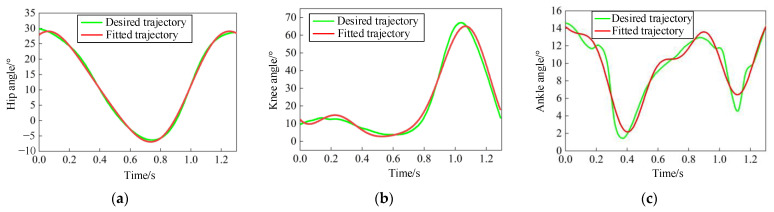
Fitting curve of the gait trajectory. (**a**) Fitting curve of the hip joint; (**b**) Fitting curve of the knee joint; (**c**) Fitting curve of the ankle joint.

The hip flexion-extension displacement trajectory is fitted using a second-order Fourier function in Equation (1), with the fitted curve shown in Figure 14a.

(1) $$\begin{array}{l} \theta_{11}=\;10.84+15.29\cos\left(5.265t\right)+8.67\sin\left(5.265t\right)\;\\ \;\;+1.789\cos\left(10.53t\right)-0.7852\sin\left(10.53t\right) \end{array}$$

Then, a third-order Fourier function is employed to fit the curve of the knee joint flexion-extension displacement trajectory in Equation (2), with the fitted curve presented in Figure 14b.

(2) $$\begin{array}{l} \theta_{12}=22.04+8.71\cos\left(4.74t\right)-22.22\sin\left(4.74t\right)-14.08\cos\left(9.494t\right)\\ \;\;-5.127\sin\left(9.494t\right)-4.268\cos\left(13.422t\right)+4.408\sin\left(13.422t\right) \end{array}$$

The ankle joint dorsiflexion/plantarflexion displacement trajectory was finally fitted using a fourth-order Fourier function in Equation (3), and the fitting curve is shown in Figure 14c.

(3) $$\begin{array}{l} \theta_{13}=10.07+1.571\cos\left(4.368t\right)-2.073\sin\left(4.368t\right)+3.702\cos\left(8.736t\right)\\ \;\;+1.541\sin\left(8.736t\right)+0.2607\cos\left(13.104t\right)-0.06254\sin\left(13.104t\right)\\ \;\;-1.4\cos\left(17.472t\right)-1.082\sin\left(17.472t\right) \end{array}$$

### 3.5. Digital Model Accuracy Verification

**Figure 15:**
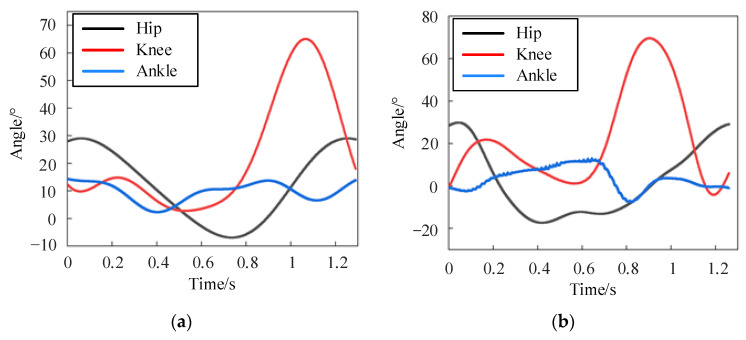
(**a**) Gait trajectory of the digital models; (**b**) Gait trajectories in the CGA database.

Figure 15a shows the trajectory of the standard gait pattern model obtained through fitting, and the accuracy of the digital model is verified through the CGA gait data trajectory from Curtin University of Technology in Australia, as shown in Figure 15b. The comparison reveals consistent movement trends between the two, indicating that the digital model obtained can serve as the desired joint trajectory for lower limb gait motion.

## 4. Gait Trajectory Tracking

### 4.1. Dynamic Modeling of the MHLRR

**Figure 16:**
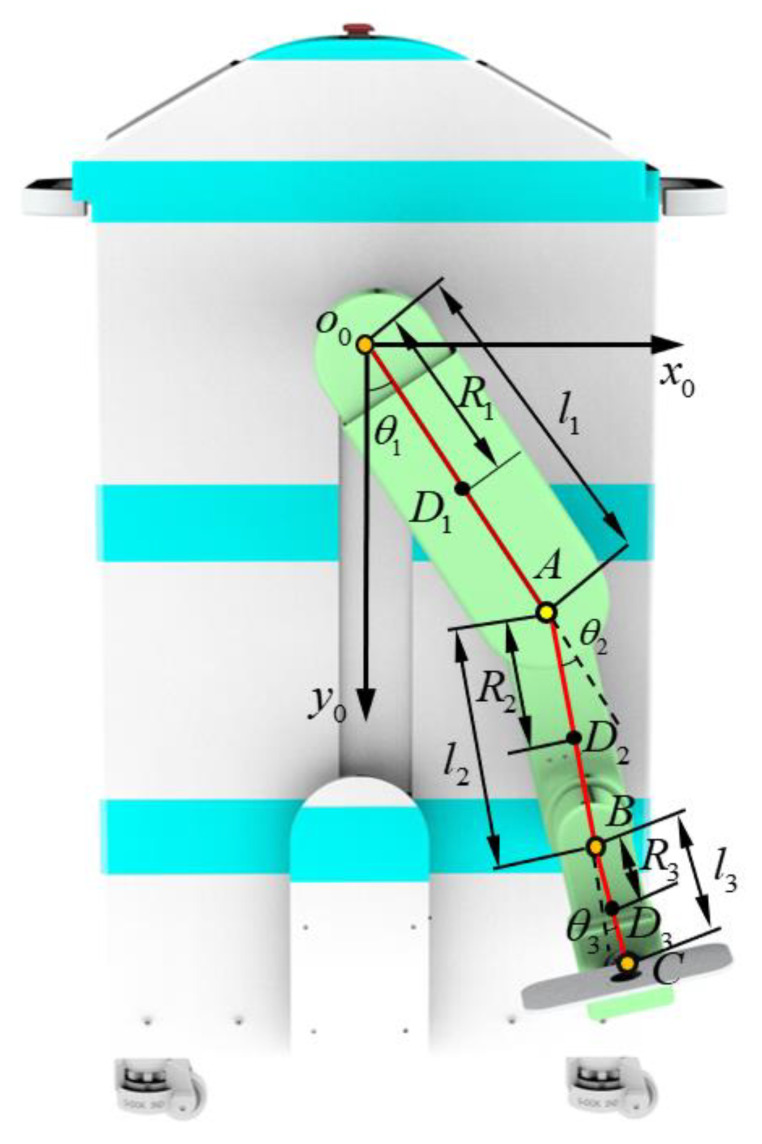
The coordinate system of the leg orthosis of the MHLRR.

The dynamics model of the lower limb training module is the theoretical basis for adaptive iterative learning trajectory tracking control. A model of the MHLRR is established, and the coordinate system is shown in Figure 16. *O*_0_, *A*, *B*, and *C* represent the hip joint, knee joint, ankle joint, and the end point of the foot, respectively. *D*_1_, *D*_2_, and *D*_3_ represent the centers of mass of the thigh, calf, and foot, respectively. *m*_1_, *m*_2_, and *m*_3_ represent the weight of the thigh, calf, and foot, respectively. *l*_1_, *l*_2_, and *l*_3_ represent the simplified link lengths for the thigh, calf, and foot, respectively. *R*_1_, *R*_2_, and *R*_3_ represent the distance between the centers of mass of the thigh, calf, and foot relative to the hip, knee, and ankle joints, respectively. *θ*_1_, *θ*_2_, and *θ*_3_ represent the angular displacement of the hip, knee and ankle, respectively.

The Lagrangian function and the dynamics equations can be obtained as

(4) $$\left\{\begin{array}{l} L(\theta ,\dot{\theta })=E_{k}(\theta ,\dot{\theta })-E_{P}(\theta )\\ \tau_{i}=\frac{d}{\mathrm{dt}}\frac{\partial L(\theta ,\dot{\theta })}{\partial {\dot{\theta }}_{i}}-\frac{\partial L(\theta ,\dot{\theta })}{\partial {\dot{\theta }}_{i}}=\frac{d}{\mathrm{dt}}\frac{\partial E_{k}(\theta ,\dot{\theta })}{\partial {\dot{\theta }}_{i}}-\frac{\partial E_{k}(\theta ,\dot{\theta })}{\partial \theta_{i}}+\frac{\partial E_{p}(\theta )}{\partial \theta_{i}} \end{array}\right.$$

where *L* represents the Lagrange function, *E_k_* represents the total kinetic energy, and *E_p_* represents the total potential energy. Additionally, *τ_i_* represents the driving torque acting on the *i*’th link, and the potential energy *E_p_* only depends on the generalized position. Moreover, ***θ*** represents the angle vector matrix, $\dot{\theta }$ represents the angular velocity vector matrix, and $\ddot{\theta }$ represents the angular acceleration vector matrix. The dynamic equation can be expressed as

(5) $$\tau +f=M(\theta )\ddot{\theta }+C(\theta ,\dot{\theta })\dot{\theta }+G(\theta )$$

where ***M***(***θ***) represents the inertial matrix, $C(\theta ,\dot{\theta })$ represents Coriolis and centrifugal forces matrix, ***G***(***θ***) represents the gravity and friction matrix, ***f*** represents the external disturbance matrix, and ***τ*** represents the torques and forces applied at each joint.

### 4.2. Controller Design for Adaptive Trajectory Tracking

**Figure 17:**
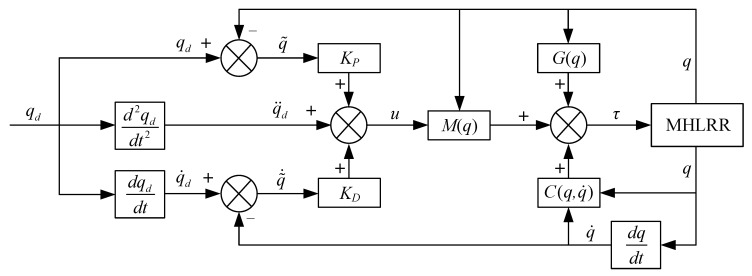
Control block diagram of iterative learning.

To suppress the influence of factors such as dynamic model parameters and external disturbances on the modeling accuracy, an adaptive iterative learning control [31] is employed, as shown in Figure 17.

Considering the impact of external disturbances on the dynamic model, the dynamics of the MHLRR can be expressed as

(6) $$\tau_{k}(t)+f_{k}(t)=M(q_{k}(t)){\ddot{q}}_{k}(t)+C(q_{k}(t),{\dot{q}}_{k}(t)){\dot{q}}_{k}(t)+G(q_{k}(t)),$$

where, t represents the time, and the nonnegative integer k represents the iteration number. $q_{k}$, ${\dot{q}}_{k}$ and ${\ddot{q}}_{k}$ represent the joint position, the joint velocity, and the joint acceleration vectors, respectively, at the k time iteration. $M(q_{k})$ represents the inertia matrix, $C(q_{k},{\dot{q}}_{k})$ represents the matrix of Coriolis and centrifugal forces, and $G(q_{k})$ represents the gravity matrix. $\tau_{k}$ represents the input matrix of the torques and forces applied to each joint. Moreover, $f_{k}$ represents the unmodeled dynamics and other external disturbances.

An adaptive control law [34] is applied for the MHLRR to adjust the switching gain, which adaptively estimates the uncertainty and disturbance terms.

(7) $$\tau_{k}(t)=K_{P}{\tilde{q}}_{k}(t)+K_{D}{\dot{\tilde{q}}}_{k}(t)+{\hat{\delta }}_{k}(t)\mathrm{sgn}({\dot{\tilde{q}}}_{k}(t))$$

(8) $${\hat{\theta }}_{k}(t)={\hat{\theta }}_{k-1}(t)+\gamma {\dot{\tilde{q}}}_{k}^{T}(t)\mathrm{sgn}({\dot{\tilde{q}}}_{k}(t))$$

${\tilde{q}}_{k}(t)=q_{d}(t)-q_{k}(t)$ and ${\dot{\tilde{q}}}_{k}(t)={\dot{q}}_{d}(t)-{\dot{q}}_{k}(t)$. Moreover, $q_{d}(t)$, ${\dot{q}}_{d}(t)$ and ${\ddot{q}}_{d}(t)$ represent the reference trajectory, the first and the second time-derivative, respectively. The matrix $K_{P}$ and $K_{D}$ are symmetric positive definite. $\mathrm{sgn}({\dot{\tilde{q}}}_{k})$ is the vector obtained by applying the signum function to all elements of ${\dot{\tilde{q}}}_{k}$. ${\tilde{\theta }}_{k}(t)=\theta (t)-{\hat{\theta }}_{k}(t)$ and ${\hat{\theta }}_{k}(t)$ is the estimated value of θ(t).

The stability of adaptive control rate is analyzed through the iterative learning of Lyapunov function, and the adaptive control rate function is convergent [34].

### 4.3. Simulation of Adaptive Trajectory Tracking

**Figure 18:**
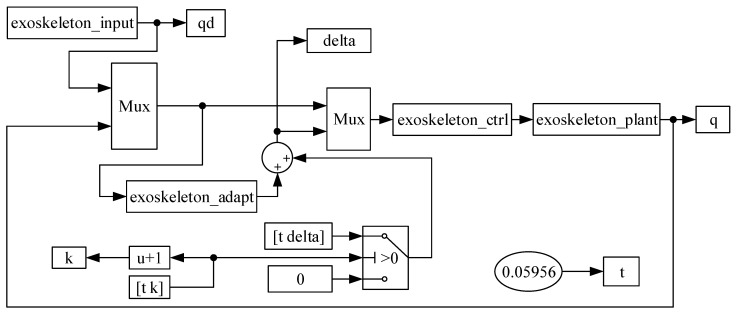
Simulation block diagram of the control system.

Figure 18 is the simulation block diagram of the adaptive trajectory control system. The exoskeleton_input is the gait-fitting trajectory input module for each joint of the MHLRR’s training module. The exoskeleton-adapt is the adaptive rate subroutine of the system control module, which estimates the uncertain items adaptively based on the switching gain. Exoskeleton-plant is the program of the controlled object, adding uncertain items and simulating the actual joint angle output. The system control subroutine module exoskeleton_ctrl simulates the actual joint angle output after adding uncertain items and disturbance items based on the fitting gait trajectory for the joint angle’s desired input.

Based on the model established in SolidWorks, the mass of the thigh link is *m*_1_ = 24.651 (kg) with the length of *l*_1_ = 0.521 (m). Moreover, the mass of the calf link is *m*_2_ = 10.135 (kg) with the length of *l*_2_ = 0.4265 (m), and the mass of the foot link is *m*_3_ = 3.908 (kg) with a length of *l*_3_ = 0.26 (m). The dynamic model parameters of the robot primarily include geometric parameters, mass properties, and joint characteristics. Firstly, we determine the geometric parameters based on the lengths of the links and the positions of the joints and establish the dynamic model according to the robot’s structure and kinematic parameters. Then, we apply controlled inputs to the robot to execute specific motion trajectories and use angular sensors and three-dimensional force sensors to collect motion data and torque information about the robot. By utilizing the collected data and employing the method of least squares to estimate the unknown parameters in the dynamic equations, the dynamic model parameters are determined through experimental data.

An adaptive iterative learning control method combining PD feedback is employed to update the input according to the tracking error of previous trajectories, progressively improving the accuracy of trajectory tracking to achieve rapid and high-precision tracking goals within a finite time interval. The parameters are set with *K_P_* = *K_d_* = 20 × 10^3^, and the number of iterations is set to 10, with 1.33 s of each simulation time.

**Figure 19:**
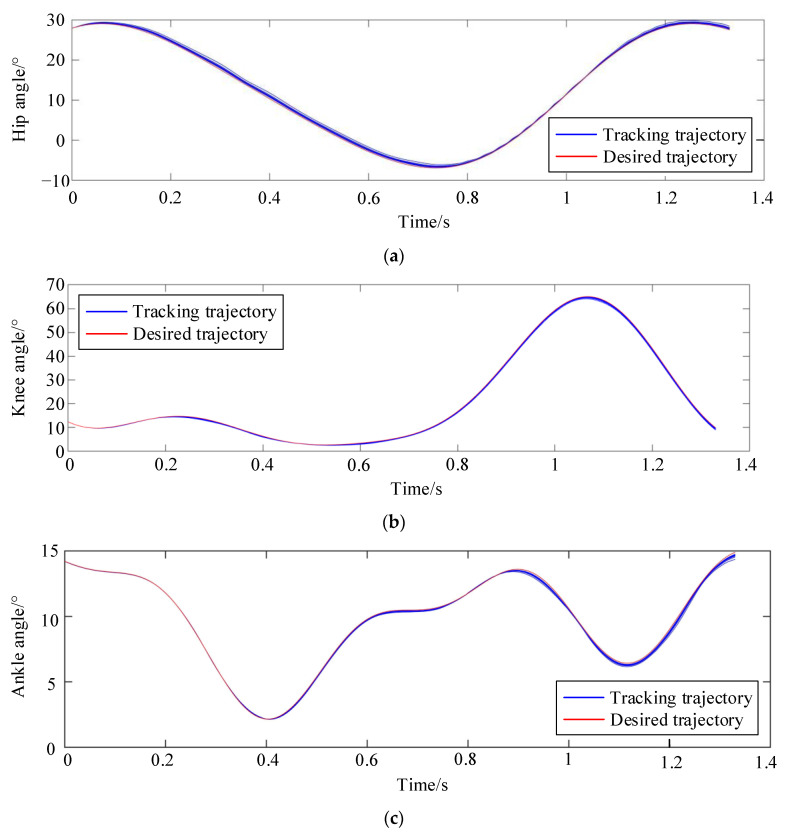
Angle tracking process with 10 iterations of learning. (**a**) Hip joint position tracking curve during 10 iterations; (**b**) Knee joint position tracking curve during 10 iterations; (**c**) Ankle joint position tracking curve during 10 iterations.

**Figure 20:**
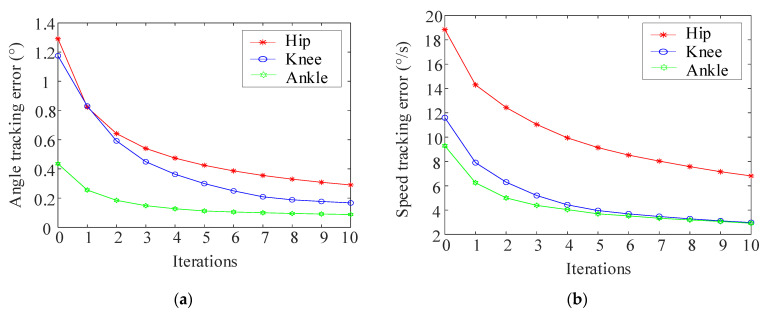
Tracking error during 10 iterations. (**a**) Angle tracking error; (**b**) Speed tracking error.

Figure 19 illustrates the joint position tracking during the 10 iterations of the iterative learning process. As depicted in Figure 20, the curve demonstrates that initial motion disturbances result in considerable trajectory tracking errors. As the iteration count increases, the output trajectory gradually converges to the desired trajectory. Additionally, the tracking error of the ankle joint’s position decreases more swiftly. Due to the relatively short total simulation time, the overall velocity tracking error remains substantial. By the iteration of k = 7, the tracking error starts to stabilize, indicating strong robustness and adaptability.

**Figure 21:**
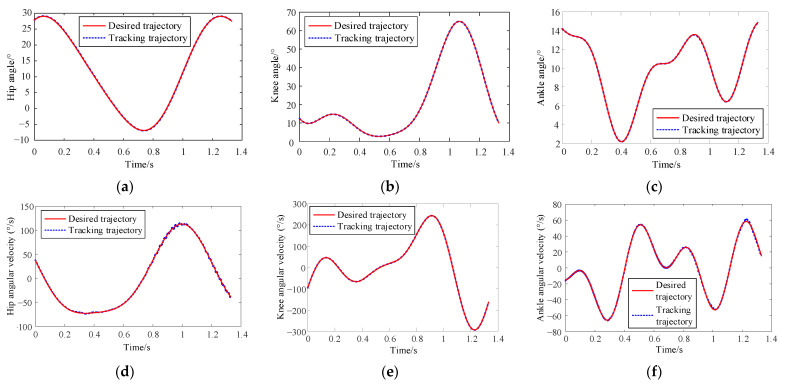
The tracking curve of joint position and velocity after 10 cumulative iterations of learning. (**a**) Hip joint position tracking after 10 iterations; (**b**) Knee joint position tracking after 10 iterations; (**c**) Ankle joint position tracking after 10 iterations; (**d**) Hip joint speed tracking after 10 iterations; (**e**) Knee joint speed tracking after 10 iterations; (**f**) Ankle joint speed tracking after 10 iterations.

Figure 21 presents the tracking results of each joint’s position and velocity after 10 iterations of learning. It is observable from the figure that the output trajectory for the positions and velocities of each joint essentially coincides with the expected trajectory. This demonstrates the effectiveness of adaptive iterative control based on switching gain control, validating that it can meet the tracking performance requirements of the MHLRR.

## 5. Experiment

### 5.1. Experimental Platform

**Figure 22:**
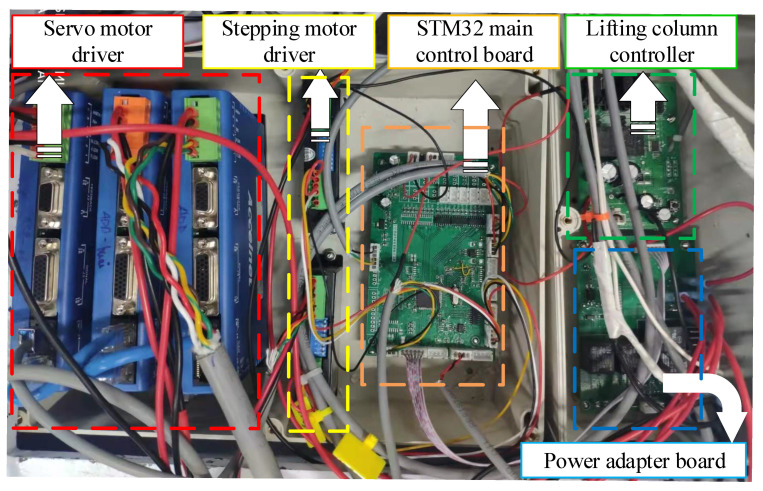
Hardware platform for motion control.

The MHLRR is controlled by the STM32 main control board through drivers and controllers, as shown in Figure 22. Among them, the servo motor driver drives the joint to achieve rehabilitation training. The stepper motor driver drives the stepper motor, which is installed inside the leg orthosis, to adjust the leg length. The lifting column driver drives the lifting motor to achieve the postural adjustment.

**Figure 23:**
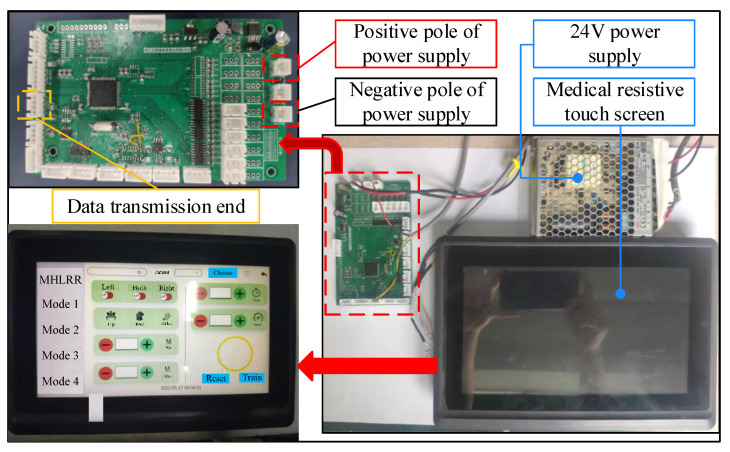
Hardware platform for human–computer interaction.

In addition, the human–machine interaction system needs to meet the electromagnetic compatibility requirements between medical devices while having a friendly and intuitive interface, making it easier for rehabilitative physicians to understand the patient’s physical ability and recovery progress. A medical resistive touch screen with a resolution of 1024 × 600 is selected as the upper computer, and virtual simulation is achieved using the built-in virtual serial port, realizing the interaction of data information with the STM32 main control board, as shown in Figure 23.

The MHLRR can actively guide patients’ limbs to move continuously in accordance with given positions and speeds through the end pedal, achieving cyclic rehabilitation training of the hip, knee, and ankle joints. Therefore, it is required that the power sources of each joint have good dynamic characteristics, such as load start-stop, smooth operation, and frequent direction changes, to avoid sudden changes in speed and acceleration during rehabilitation training. Taking all factors into consideration, the direct-current servo motor is chosen as the joint driving power source. It communicates with the STM32 main control board via the Copley driver based on the CANOPEN communication protocol to complete the transmission of relevant control commands. The parameters of each joint driving part are shown in Table 5.

### 5.2. Gait Trajectory Tracking

**Figure 24:**
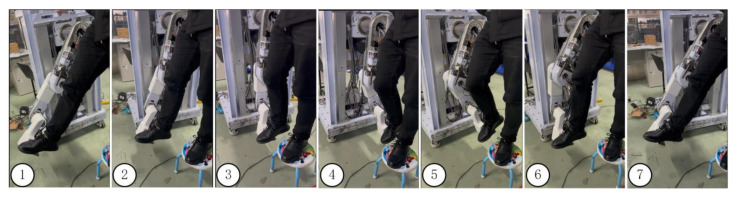
The complete cycle gait trajectory of the MHLRR.

Due to different forms of motor dysfunction in patients with hemiplegia during the later stages of recovery, combining gait trajectory digital models with the MHLRR enables more comprehensive training of the lower limb muscles, thereby improving balance and walking ability. Figure 24 shows the implementation of gait trajectory during right leg training mode using the MHLRR based on the collected gait models. The figure caption 1–7 in Figure 24, respectively, are seven illustrative diagrams of a complete gait cycle in chronological order.

**Figure 25:**
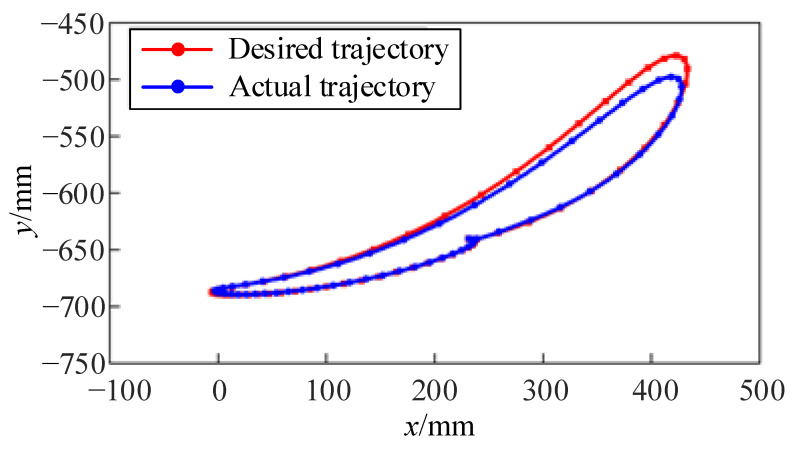
Comparison of the endpoint trajectories in gait.

Figure 25 is a comparison of the desired and actual endpoint gait trajectories in the sagittal plane. The gait trajectory starts from the maximum angular displacement of the hip joint and gradually completes the typical positions of the stance phase and swing phase. The actual end trajectory is calculated based on the joint trajectories collected by each joint and the forward kinematics of the lower limb training module. The transition of joint motion between each position is smooth, validating the rationality of the gait trajectory.

**Figure 26:**
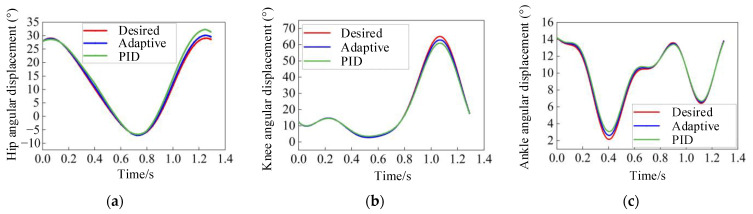
Comparison of joint angular displacement among desired trajectory, adaptive control trajectory, and PID control trajectory. (**a**) Comparison of hip joint angular displacement; (**b**) Comparison of knee joint angular displacement; (**c**) Comparison of ankle joint angular displacement.

As indicated by the gait trajectory digital model, the duration of a single gait cycle is 1.2917 s. Gait training commences at the point where the right heel first makes contact with the ground, which marks the starting position of the gait cycle. The comparison of the motion curves is illustrated in Figure 26. The adaptive control strategy based on PD feedback has significant advantages over PID control in terms of joint trajectory tracking performance. It reduces the root mean square errors of the hip joint by 1.2°, the knee joint by 0.96°, and the ankle joint by 0.215°, meeting the high-precision tracking requirements of the MHLRR.

## 6. Conclusions and Future Work

This paper proposes a multistage hemiplegic lower-limb rehabilitation robot for patients with hemiplegia in multiple stages of recovery to rehabilitate conveniently and cost-effectively with a single device. The mechanism of the MHLRR was designed, and a multi-mode limiting structure was proposed, including the hip with five modes, the knee with two modes, and the ankle with two modes. On this basis, the MHLRR can adapt to hemiplegic patients in multiple stages to train in lying, sitting, and standing positions on any training side. The gait trajectory has been collected and extracted through a motion capture device called XSENS. Then, the gait trajectory model was planned. In addition, in order to improve the high-precision tracking performance of the MHLRR, a control system for the training module based on adaptive iterative learning has been built. Based on this, the tracking performance is verified through the simulation. Finally, the experiment with the gait trajectory was completed, and the preliminary experimental trials verified the effectiveness of the gait trajectory model and its tracking performance.

In the future, further improvements are to be made to the MHLRR based on gait trajectory planning. For example, the gait trajectories can capture a wider range of subjects with different characteristics, making the model more targeted for different patients. It is also noted that the proposed function of the MHLRR needs to be tested on real patients instead of volunteers, which may expose more problems for the mechanical design and control strategy, i.e., clinical tests should be made in the future work.

## Figures and Tables

**Table 1:** The length of various body parts of the participant.

Height	Shoulder Height	Hip Height	Knee Height	Ankle Height
168 cm	138 cm	94 cm	47 cm	9 cm

**Table 2:** Sample partitioning of BP neural network.

Number	1	2	3	4	5	6
Cycle interval (s)	1.512–2.8	2.8–4.046	4.046–5.263	7.558–8.946	8.046–10.12	10.12–11.34
Number	7	8	9	10	11	12
Cycle interval (s)	14–15.25	15.25–16.42	18.8–20.08	20.08–21.27	21.27–22.49	24.97–26.19
Number	13	14	15	16	17	18
Cycle interval (s)	26.19–27.42	27.42–28.67	28.67–30	30–31.35	31.35–32.56	32.56–33.81

**Table 3:** Error with 5, 10, and 15 hidden layer nodes.

Number of Hidden Layer Nodes	Mean Absolute Error (MAE)	Mean Squared Error (MSE)	Root Mean Square Error (RMSE)
5	2.3207	7.9289	2.8158
10	2.2851	7.6949	2.7740
15	2.3101	8.0051	2.8229

**Table 4:** The characterization coefficient of fitting effect.

Joint Angular Displacement	Fitting Times	Variance (SSE)	Root-Mean-Square (RMSE)	Correction Coefficient (R-Square)
Hip	2	117	0.6203	0.9977
Knee	3	314.4	1.02	0.9976
Ankle	4	247.1	0.8872	0.9416

**Table 5:** The parameters of the joint drive part.

Joint	Hip	Knee	Ankle
Motor Model	SMP8024	NBL9040	EC60
Rated Power (W)	382	335	150
Rated Torque (N·m)	1	0.9	0.401
Rated Voltage (V)	24	24	24
Rated Speed (rpm)	3650	4700	3480
Reducer Model	HSG40	HSG32	HSG20
Reduction Ratio	100	100	100
Rated Torque (N·m)	345	178	52

## Data Availability

The data presented in this study are available on request from the corresponding author.
